# Conventional and Advanced Magnetic Resonance Imaging Biomarkers of Multiple Sclerosis in the Brain

**DOI:** 10.7759/cureus.79914

**Published:** 2025-03-02

**Authors:** Othman I Alomair

**Affiliations:** 1 Radiological Sciences Department, College of Applied Medical Sciences, King Saud University, Riyadh, SAU

**Keywords:** black hole lesions, brain atrophy, central vein signs, enhanced ms lesions, imaging biomarkers, magnetic resonance imaging, mcdonald criteria, ms phenotypes, multiple sclerosis, neurological disabilities

## Abstract

Multiple sclerosis (MS) is a heterogeneous disease, and each MS patient exhibits different clinical symptoms that are reflected in their magnetic resonance imaging (MRI) results. Each MS lesion should be interpreted carefully and evaluated in conjunction with a clinical examination. MRI plays a major role in evaluating how MS lesions are aggregated in the central nervous system and how they change over time. There are several conventional MRI biomarkers of MS that could be utilized to evaluate each MS phenotype. MRI is useful for clinical decisions, aiding in the determination of disease-modifying treatment or disease prognosis. Despite its higher sensitivity, MRI provides low specificity due to the heterogeneity of MS lesions. However, advanced MRI biomarkers show promise in terms of defining MS lesions, as each imaging biomarker correlates differently with the clinical scenario of each MS phenotype. The aim of this review is to summarise the current state of MRI biomarkers for MS in the brain and how they relate to neurological disabilities.

## Introduction and background

**Figure 1:**
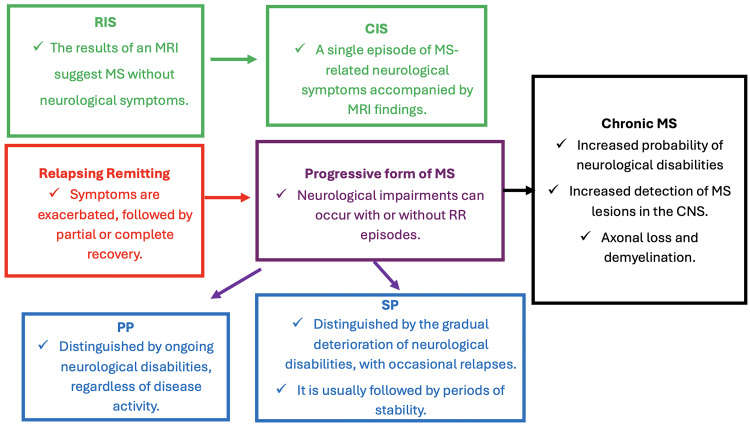
A simple chart diagram to explain the various MS phenotypes. MS: multiple sclerosis, PP: primary progressive, RIS: radiological isolated syndrome, CIS: clinically isolated syndrome, RR: relapse-remitting, SP: secondary progressive

Multiple sclerosis (MS) is an immune disease that attacks nerve fibers and results in demyelination, axonal damage, and severe axonal loss. This dramatically affects the central nervous system (CNS) and causes clinical complications, such as imbalance, blurred vision, difficulty moving, and other severe symptoms [1]. Each patient with MS exhibits different clinical symptoms, which should be integrated with imaging findings to determine the MS phenotype. MS clinical diagnoses include clinically isolated syndrome (CIS) and primary progressive (PP), relapsing-remitting (RR), and chronic MS [2]. It is a complex disease that typically begins with a clinically isolated syndrome (CIS). This phenotype is distinguished by the patient's initial neurological signs of MS, which are transient but correspond with MRI findings. Another MS subtype is radiological isolated syndrome (RIS), which is characterized by MRI findings in the absence of neurological symptoms. All these early MS phenotypes can progress to definite multiple sclerosis, with significant differences between individuals. On the other hand, RR, as the name implies, refers to an exacerbation of symptoms followed by partial or complete recovery [2]. While both PP and SP (secondary progressive) are forms of MS progression, they are distinguished by a higher prevalence of neurological impairments in the absence of relapsing-remitting episodes. PP is distinguished by the steady progression of neurological disabilities with or without disease activity. SP is distinguished by a progressive deterioration of neurological disabilities, with occasional relapses. This is typically followed by periods of stability. Chronic MS is characterized by an increased likelihood of neurological disabilities and a greater detection of MS lesions in the CNS, which are marked by axonal loss and demyelination [1-3]. Figure 1 depicts a simple chart diagram to explain the various MS phenotypes.

Evaluating MS accurately requires evidence, such as clinical or imaging findings and laboratory results [2,3]. The McDonald criteria serve as a set of guidelines to assess the progression of MS diagnosis and treatment. These criteria are based on MRI findings, clinical neurological assessments, and cerebrospinal fluid (CSF) analysis tests. These criteria have played a major role in evaluating the progression of MS and whether patients are receiving the appropriate treatment [4]. These criteria demonstrate how MS lesions localize and affect different CNS areas, either in the brain or spine, which is known as dissemination in space (DIS), and how MS lesions evolve over time, which is known as dissemination in time (DIT) [5,6]. These criteria were introduced in 2001 and have been revised periodically, with the most recent version published in 2017 [5,7]. Regarding diagnosis, it is important to define the term MS lesion, as it recurs in our research and is a common term in radiologist reports that is open to interpretation. A lesion is a spot or an area in a magnetic resonance image that differs in appearance from the intact area around it. Lesions can be classified based on conventional imaging into T2 hyperintense or bright spot lesions, T1 hypointense or black hole lesions, and enhanced lesions [3,6]. The confirmed observation of two or more focal demyelination lesions in different CNS areas is suggestive of DIS. An enhanced lesion, which evolves or alters over time, could also be an indicator of DIT [7]. According to the Multiple Sclerosis Trust website [8], the updated McDonald criteria for 2024 have not yet been published. CVS and paramagnetic rim lesions were identified as new image biomarkers. They can be identified by central blood vessels or an iron ring around the edges of the lesions. Furthermore, white blood cells produce kappa-free light chains. They are classified as an immune response. These lab biomarkers are comparable to oligoclonal bands in CSF and have the potential to aid in MS diagnosis. In the 2024 version, the optic nerve is listed as a fifth typical MS location, indicating the presence of DIS. Furthermore, DIT is not required if the MS patient has four or five MS lesions in the CNS [8].

MS is diagnosed using a variety of clinical assessments, including patient history documentation, a neurological examination, and various paraclinical diagnostics. The combination of neurological symptoms and the ambiguity of distinct illness biomarkers make reaching a definitive conclusion difficult. The Expanded Disability Status Scale (EDSS) and the Kurtzke Functional Neurological Status (FSS) are two examples of neurological assessments. These tests are critical for determining the degree of deterioration in impairments and neurological functions. MRI, on the other hand, is the preferred imaging modality for assessing MS lesions because it is non-invasive and can provide detailed visualization of central nervous system anatomical features. Nonetheless, additional clinical assessments, such as cerebrospinal fluid analysis using oligoclonal bands (OCBs), are carried out. It is a distinguishing feature of MS and helps to differentiate it from other neurological disorders. Combining MRI and CSF studies would improve understanding in an MS diagnosis, both structurally and functionally [1-8].

MRI is an essential tool for confirming both DIS and DIT [5]. It is a non-invasive imaging technique that can be used to evaluate brain structures and functions and to demonstrate patterns that are very common in MS. MRI offers a variety of image contrast options as well as facilitating image acquisition in different anatomical sections, which could be integrated to demonstrate how MS lesions are located within the CNS [3]. To evaluate MS lesions accurately, the routine MRI protocol should include at least the following imaging sequences: pre- and post-T1-weighted images (WIs), T2-WIs, and fluid-attenuated inversion recovery (FLAIR) images [1,3].

MS lesions usually consist of inflammatory edema, demyelination, and the coexistence of either axonal damage or loss. These lesions commonly aggregate in certain parts of the central nervous system (CNS), such as the periventricular white matter, juxtacortical and infratentorial regions, optic nerve, and spinal cord, which are the most typical locations for MS lesions. They can be indicated by bright spots or hyperintense signals on T2-WIs due to the prolongation or lengthening of the T2 relaxation time. FLAIR imaging sequences have assisted in identifying more MS bright spots because they suppress cerebrospinal fluid and augment the image contrast, thus depicting MS lesions better compared with T2-WIs [1,2].

T2 WI and FLAIR are standard, integral components of the clinical diagnosis of DIS. T1-WIs are frequently acquired pre- and post-gadolinium (Gd) contrast. T1-WIs are utilized to identify black hole lesions, which could be interpreted as severe axonal damage. In contrast, post-Gd contrast T1-WIs can indicate disease activity by depicting Gd-enhanced MS lesions that break down the blood-brain barrier (BBB). Imaging sequences are routinely utilized for MS patients [3,4]. However, while they are highly sensitive, they are less specific due to the heterogeneous nature of MS lesions, which are characterized by axonal damage, loss, and a mixture of demyelination and remyelination [6].

On the other hand, better MS imaging biomarkers are being developed to improve specificity in the interpretation of MS lesion heterogeneity. When compared to T2 WI or FLAIR, double inversion recovery (DIR) detected cortical and juxtacortical MS lesions more effectively. Furthermore, diffusion tensor imaging (DTI) assesses normal-appearing white matter (NAWM), which can distinguish MS from other mimic diseases. Central vein signs (CVSs) are a distinctive MS imaging feature [1-8].

In this review, routine MRI sequence protocols and conventional brain imaging biomarkers are outlined and reviewed. In addition, advanced imaging techniques are briefly presented, and examples of developed imaging biomarkers are introduced with a discussion of their diagnostic values and limitations based on current clinical practice. This paper is a narrative literature review where PubMed, Google Scholar, and ScienceDirect databases were used to find references to MRI biomarkers of MS in the literature. The suggested terms include "multiple sclerosis", “black hole lesions, or T1 hypointense lesions", “central vein sign", “clinical isolated syndrome", “primary progressive", “MRI protocols of MS", "brain", “MS biomarker", “secondary progressive”, “MS-enhanced lesions”, “cortical lesions”, “hyperintense MS lesions”, and “Artificial intelligence in MS”. Only papers written in English were considered for review.

## Review

Routine MRI brain protocols for MS

According to the 2017 updated version of the McDonald diagnostic criteria for MS [7], the recommended MRI protocol should include double-echo proton density (PD)/T2-WIs using fast spin echo (FSE) sequences, in addition to axial and sagittal T2-weighted FLAIR images, and axial post-contrast T1-WIs. MRI is best performed using 3 Tesla (3T) MRI scanners and via 3D acquisition, offering better lesion detection and better anatomical realignment, thus assisting in spotting new lesions and easily monitoring lesion progression over time in different brain anatomical locations [1,9,10].

Three-dimensional FLAIR acquisition is currently the most widely employed imaging technique due to its high sensitivity. If 3D FLAIR is not available or not readily accessible in some imaging centres, 2D FLAIR can be performed with thinner slices of less than 3 mm, without a slice gap [1,3,10].

Magnetic field strength plays a crucial role in boosting imaging quality. A scanner with a higher magnetic field strength provides a higher spatial resolution within a shorter scan time than an MRI scanner with a lower magnetic field strength [11,12]. For example, a 3T MRI scanner increases the detection rate for MS lesions compared with a 1.5T scanner [12,13]. However, it does not assist in early MS diagnosis; therefore, 1.5T scanners are the optimum choice for MS diagnosis if they can achieve a high signal-to-noise ratio (SNR) and 1 mm isotropic resolution [14,15]. Moreover, scanners weaker than 1.5T are not recommended [1,3]. On the other hand, ultra-high-field MRI scanners, such as 7T units, are currently utilised for research purposes, showing promise because they can detect more challenging cortical lesions (CLs) [11,16,17]. However, they are not clinically applied due to the difficulty in image interpretation because of the ultra-high magnetic field altering the tissue relaxation time, which consequently affects the image contrast. In addition, 7T scanners are not offered in most MRI clinical centres; therefore, 1.5T units are still recommended by most MS imaging guidelines [1,3,7].

The deposition of Gd, how long it stays in the body, and how frequently it can be utilised are major safety concerns [18]. This has led to new recommendations and updated regulations from the American Food and Drug Administration (FDA), the Consortium of Multiple Sclerosis Centres (CMSC), and Magnetic Resonance Imaging in Multiple Sclerosis (MAGNIMS) organisations to limit the administration of Gd for evaluating MS patients. Despite all these safety measures, Gd is still necessary and useful during the early evaluation and investigation of lesions to demonstrate how MS lesions are evolving and changing over time [19]. Previous studies demonstrated how the higher sensitivity of triple and double doses of Gd assisted in detecting more MS lesions compared with a single dose (0.1 mmol/kg); however, current recommendations emphasise the utilisation of a single dose due to the safety concerns [1,9,20,21].

CIS is known as the first stage or sign of MS, whereby the patient experiences a single episode of neurological symptoms. In general, it is not a definitive diagnosis because not all patients will develop a confirmative MS presentation. This increases the demand for patient follow-up via serial MRI scans [22,23]. T2-WIs are recommended for detecting and revealing new MS lesions [3,23] as they can reveal bright lesions within different brain areas and consequently lead to a confirmed diagnosis of MS [1]. Several studies have demonstrated the significance of serial T2-WIs, which are proposed as imaging markers for monitoring lesion change over time, and there has been a subsequent reported increase in sensitivity (74%) and specificity (92%) [24]. In addition, a 20-year longitudinal study highlighted the significance of measuring lesion volumes using T2-WIs, reporting a three-fold increase in lesion volumes in MS patients who had developed secondary progressive compared with RR phenotypes [23].

Currently, FLAIR imaging sequences are strongly recommended in routine brain MRIs and have become a core imaging pulse sequence for multiple sclerosis, either via 2D or 3D imaging acquisition [25,26]. The periventricular area is a typical MS lesion location, and the aim of this imaging sequence is to suppress all CSF signals so that MS lesions adjacent to the CSF can be visualised and detected more easily. A recent study reported that 3D FLAIR is superior to 2D FLAIR in detecting more MS lesions due to its higher signal-to-noise (SNR) and contrast-to-noise ratio (CNR) [26]. This was shown to assist in improving diagnostic accuracy due to the enhancement of the lesion visibility, and consequently, this could play a major role in monitoring disease activity, evaluating the disease-modifying treatment (DMT), and how the treatment is adjusted [27].

Diagnostic Value of T2-WIs and Inversion Recovery Sequences

T2-WIs are an integral component of the MS imaging protocol [28]. To fulfil the McDonald criteria for MS and determine DIS, T2-WIs must depict one or more T2 hyperintense lesions in at least two of the most common typical MS locations. In addition, T2-WIs play a significant role together with T1-WIs in determining DIT; for example, in a follow-up MRI, if a new hyperintense T2 lesion is detected, it would be enough to demonstrate MS is evolving or changing over time. It is therefore better to identify enhanced MS lesions to fulfil DIT, but this may not be required [28,29].

The use of inversion recovery pulse sequences is a well-designed imaging technique that can be utilized to suppress signal intensity by adjusting the inversion time (TI). This parameter could be altered based on the T1 relaxation time of the suppressed tissue [30]. In MS imaging, it is very common to suppress the CSF signal of the brain. This will facilitate the better evaluation of MS lesions adjacent to brain ventricles, which is a typical MS location [31,32]. Currently, double inversion recovery (DIR) sequences are being introduced, which allow for the suppression of white matter (WM) and CSF. This is based on the selected TI as an imaging parameter, which depends on the T1 relaxation time of the suppressed tissue. Inversion recovery sequences are usually acquired with FSE sequences either via 2D or 3D acquisition. Recent studies reported an increase in the sensitivity for detecting CLs using DIR sequences [33,34]. CLs were detected in early MS stages, and they loaded more lesions as the MS progressed and evolved over time [35].

Hyperintensity lesions: MS lesions are usually depicted with bright signal intensity on either T2-WIs or FLAIR imaging sequences [3]. MS tends to affect both brain hemispheres symmetrically, but it could be asymmetrical in the early stages of the disease. Common locations include periventricular white matter areas, the corpus callosum, and juxtacortical areas. In addition, these lesions can be seen within the infratentorial areas, in either the pons or cerebellum. The shape of the MS lesion is commonly circular or oval, with a generic size between 3 and 5 mm in its longest dimension, with variation depending on the anatomical location; for example, a less than 3 mm lesion adjacent to the floor of the fourth ventricle is considered abnormal [6,29].

Slice thickness is an important imaging parameter when determining the size of a detected lesion. If the slice thickness is less than 3 mm, the MS lesion can be diagnosed in a single slice. In contrast, with thicker slices, hyperintense lesions should be demonstrated in at least two consecutive slices. This is paramount to exclude other hyperintensities due to other abnormalities [29]. Lesion load is defined as the number of T2 hyperintense lesions, which are detected in the WM or other common MS locations. It is also a potential imaging biomarker for estimating the developing physical disabilities in either CIS, early relapsing MS, or a secondary progressive course. Despite this, the correlations between lesion load and prognosis were only modest [23,36]. A higher infratentorial lesion load resulted in a better prediction of long-term disabilities in either CIS or RR MS patients, which means that location does matter prognostically [37-40].

The pons is a structure in the infratentorial region or brainstem. It is involved in facial sensations and has control centres that dictate how one’s face and eyes move. It also acts as a coordinating centre, as it communicates motor information sent from the brain to the feet as well as sensory information sent from the feet to the brain [38]. If there is an MS lesion within the pons, the patient typically experiences severe neurological symptoms [37]. This emphasizes the importance of serial MRI scans to evaluate the areas of the brain affected by MS lesions because neurological symptoms vary based on which CNS structures are impacted by hyperintense MS lesions.

In addition to evaluating T2 hyperintense lesion loads, CLs, which are distinct features of MS, are potential clinical imaging biomarkers during the early stages of MS, in addition to having a role in confirming the conversion of CIS to definitive MS [41]. CLs are also used to differentiate between MS and conditions that mimic MS [35,42]. However, there are still various challenges in imaging CLs due to their smaller size and cortical anatomical location. The development of 7T MRI scanners and the availability of new imaging sequences, such as gradient echo sequences (GRE) sequences with generated phase signals and phase-sensitive inversion recovery (PSIR) sequences, have resulted in the detection of more categories of CLs, such as subpial, intracortical, and leukocortical lesions [43-46]. 

Diagnostic Value of T1-Weighted Imaging

Demyelination and axonal loss are major features of MS, and these pathological changes cause the prolongation of the T1 relaxation time. Two-dimensional T1-weighted images are acquired with a short repetition time and short echo time using spin echo sequences because this method is feasible with a reasonable scan time. However, fast spin echo (FSE) is preferred for acquiring 2D T1-WIs because it offers a higher spatial resolution with a much shorter scan time [31,47]. Currently, 3D T1-WIs use substituted 2D sequences, which are usually acquired using FSE or gradient echo (GRE) sequences. Under high-magnetic-field conditions, 3D T1-WIs offer a high isotropic resolution, a high SNR, and a significant improvement in image contrast. This is usually acquired via the pre- and post-contrast administration of Gd and enables essential evaluations like brain volumetric measurements and assessments of disease activity, which rely on how many MS lesions were detected in post-contrast T1-WIs. In addition, pre-contrast T1-WIs are utilised to evaluate black hole or hypointense lesions, which are surrogate imaging biomarkers of severe tissue damage in MS [1,6].

Brain atrophy: All adults have a slow rate of brain shrinkage due to ageing changes [48]. People with MS are at risk of faster annual brain shrinkage [49,50]. Brain atrophy is a significant MS feature characterised by a measurable decrease in brain volume, with recent studies demonstrating an annual brain volume loss of 0.5 to 1.35 % [49,50]. This percentage is higher than the brain volume reduction usually observed in a normal population due to the normal ageing process [49,50]. 3D T1-weighted images are obtained at high resolution and are widely used in clinical practice to estimate brain volume measurements [51-53]. There are a variety of software and post-processing tools available for measuring brain volume or segmentation in longitudinal studies. Examples include FSL, FreeSurfer, and SynthSeg, but this list is not exhaustive [52-55]. Another software, NeuroQuant® (Cortechs.ai, San Diego, USA) was used to quantify 3D voxel-based brain morphometry. This was useful in demonstrating volume reductions in grey matter and cortex, which correlate with neurological disabilities [52].

Brain volumetric measurement, as a total or for specific CNS anatomical structures, plays an essential role in defining the development of neurological disability, such as cognitive impairment [56]. Whole-brain atrophy was reported in various studies as a surrogate imaging biomarker for MS prognosis [57,58]. Evaluating how the atrophy accelerates is a critical factor in defining the progression of MS, as it is clinically strongly correlated with cognitive decline and physical disability [58-60]. A 6- and 12-year follow-up study of brain atrophy as a predictor for disabilities and degrading cognitive function demonstrated a significant correlation after 6 years but not after 12 years. In contrast, another large multicentre follow-up study showed whole-brain atrophy as a predictor of the progression of disabilities within 10 years [58].

Atrophy was not only observed in the total brain volume [61] but was also demonstrated in specific CNS anatomical areas such as the ventricles, grey matter, and corpus callosum [57-59,63,64]. Atrophy in these mentioned anatomical areas is positively correlated with neurological disabilities. However, atrophy in the basal ganglia, medulla oblongata, and limbic structures was inconsistently related to disabilities in MS patients [65-67]. In contrast, atrophy in the WM was not strongly correlated with MS progression, and this relationship was more complex. For example, a study reported that cortical atrophy is better than WM atrophy in predicting disabilities [68]. On the other hand, a different study demonstrated a significant correlation between increased WM volume loss and MS progression [69]. Therefore, the atrophy of WM is not a conclusive imaging biomarker according to a systematic review of recent studies [64]. Total brain volume loss and atrophy in any brain anatomical structure are common features in most MS phenotypes [70], but they should be considered alongside other imaging biomarkers, such as lesion load or the number of active lesions [58].

Enhanced MS lesions: In addition to brain volume measurement, acquiring post-Gd 2D or 3D T1-WIs is an essential tool for monitoring and defining active inflammation and demyelination within the CNS [71]. Gd is a traditional contrast agent with paramagnetic properties, and once this agent is administered, it will not normally cross the BBB unless it is compromised due to active inflammation. It is commonly observed as a hyperintense signal on T1-WIs and is known as a Gd-enhanced MS lesion. This is a key and integral imaging biomarker of MS progression over time [1,3]. Gad reduces the T1 relaxation time, resulting in increased signal intensity. This is commonly seen when Gd accumulates within normal brain anatomical structures such as the pituitary gland, choroid plexus, and nasal mucosa. Recent studies have demonstrated Gd deposition in brain structures such as the dentate nucleus and globus pallidus [72,73]. However, Gd is still used as a contrast agent, and more research is needed to understand why it is retained in the brain. On the other hand, Gd is critical for monitoring the progression of MS. If there are MS lesions, they will shorten T1 relaxation time and appear as a hyperintense signal on T1-WIs, which is known as a Gd-enhanced MS lesion.

Recent recommendations on the use of MRI in MS patients suggest including Gd-based contrast agents on the baseline MRI [1,3]. This is necessary to confirm changes in the disease over time. In addition, it assists in MS diagnosis by evaluating enhancement patterns, which is valuable in treatment decisions based on lesion activity; consequently, MS phenotypes can thus be determined [3,21]. It has been recommended to acquire an MRI with Gd during the first year after DMT is given; this is very important if the DMT is interferon beta or glatiramer acetate. This kind of treatment should be monitored closely because it is unlikely to reduce the number of enhanced lesions when compared with other treatments [74]. In addition, if the T2-WIs demonstrate a large lesion burden characterised by diffuse and confluent MS lesions, then Gd should be administered to predict new disease activity, which is difficult to evaluate using only T2-WIs. In contrast, the use of Gd will not be required for demonstrating a change in disease activity within one year if the MS patient had a recent MRI scan. Additionally, Gd is unnecessary 3 to 6 months following the DMT or if a short follow-up was performed within 6 months. For female MS patients, Gd should be avoided in all cases during pregnancy or breastfeeding [3,7].

Black hole or T1 hypointense lesions: MRI can assist in detecting a variety of MS lesions [6,29]. Pre- and post-contrast T1-WIs should be acquired to facilitate a comparison between different MS lesions. Black hole or T1 hypointense lesions are usually depicted in pre- or post-contrast T1-WIs and appear with very low signal intensity, whereas they appear as hyperintense in the corresponding T2-Wis [6,75,76]. Black hole lesions are represented as holes, which are areas in which the inflammation is so intense and significant that it affects the brain tissue and consequently causes severe tissue damage [77]. A T1 black hole is an early predictor of long-term disability compared with bright focal lesions detected using T2-WIs [78]. It is becoming common practice to evaluate the size and signal intensity of the black hole lesion to determine how the MS is progressing [75,79].

Black hole lesions originated as enhanced MS lesions, which could be either short-lived, lasting for 6 to 12 months, or permanent, consequently causing irreversible demyelination and severe tissue damage [6,47]. It is essential to evaluate the age of these lesions and the change in signal intensity because the transient black hole lesions are characterised by oedema and a mixture of demyelination and remyelination of the nerve axons. Short-term black hole lesions occur coincidentally with enhanced lesions in T1-WIs, whereas chronic black hole lesions are more persistent and show close relatedness with brain atrophy [47,80,81]. In a previous study, these lesions were found to become permanent after their enhancement diminished; the study acquired T1-WIs monthly for 4 years and reported that 55.7% of transient black hole lesions became permanent and this was related to enhanced lesion activity [47].

Black hole lesion load is a potential indicator of neurological disability in either secondary progressive MS or primary progressive MS, with a higher number of black holes reported in these MS categories compared with RR MS patients [80,82]. In addition, it is recommended that the black hole lesion load be integrated with the T2 lesion loads because it increases the accuracy of predicting neurological disabilities [78,83]. This method is superior for determining the spatial distribution of the black hole lesions and how they affect different brain anatomical structures [84], which will add significant prognostic value for predicting cognitive impairment. A recent study reported a significant correlation between the black hole lesion volume observed in the frontal lobe and phonemic verbal fluency [84]. In addition, there was a significant correlation between the black hole lesion volume in the parietal lobe and attention. The same study also reported the significance of parietal and temporal black hole volume measurement in predicting impairment in nonverbal intelligence tasks [84].

Advanced MRI techniques and biomarkers

Central Vein Sign (CVS)

To accurately assess CVS, another brain imaging sequence should be carefully evaluated. T2*-WI is acquired using GRE sequences via either 2D or 3D acquisition. It is different from other imaging sequences because it requires high magnetic field homogeneity [85]. In terms of physical concepts, the T2* relaxation time is different from T2 relaxation because it occurs due to the decay of T2 and magnetic field inhomogeneities. Therefore, T2*-WI demonstrates how the tissues are magnetised to different degrees, which is known as magnetic susceptibility-weighted imaging. Therefore, it depicts the difference between tissues in their T2* relaxation time, which relies on magnetic susceptibility properties [85]. For instance, normal tissues in the brain are inherently diamagnetic; however, if there are blood products like iron, they consequently induce paramagnetic properties and cause signal voids.

**Figure 2:**
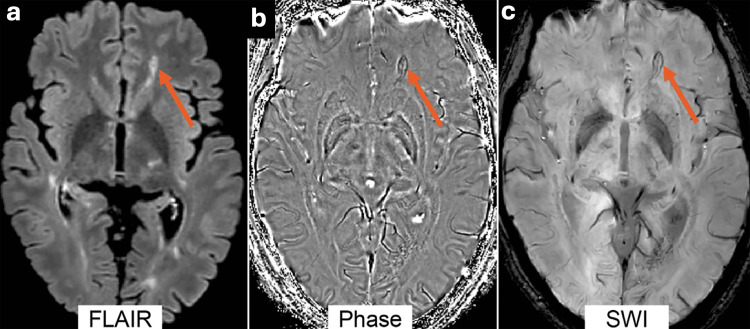
An example of hyperintense lesions shown on (a) FLAIR at 3T. The red arrow indicates one of the hyperintense lesions, which has a hypointense rim on the (b) phase image and (c) SWI. Reproduced from Rocca MA et al [3] under the terms of the Creative Commons Attribution License (CC BY 4.0).

T2*-WI is currently routine for MRI of the brain due to its higher sensitivity in cerebral haemorrhage, microbleeding, and iron deposition in the CNS [86,87]. In terms of MS diagnosis, the central vein sign (CVS) is a common MS feature that is depicted on T2*-WIs as a small vein within the MS lesions. The CVS is a promising MRI biomarker because it provides several valuable pieces of clinical information. First, it can discriminate MS from other demyelination diseases and mimics. Second, it provides higher sensitivity and specificity, which will probably increase diagnostic accuracy and become routine in future clinical practice [88,89]. The features of MS lesions are not always visualised as central veins; they could be observed differently in the basal ganglia with either an iron rim or loading in these CNS areas [90,91]. All these pathological changes can be detected and accurately diagnosed with newly developed MRI sequences, such as quantitative susceptibility mapping (QSM). While GRE images consist of magnitude and phase images, QSM relies on phase shifts that occur due to the induction of susceptibility in the magnetic field to inhomogeneity [92]. QSM provides better contrast compared with T2*-WIs; therefore, it can be applied to quantify the iron, myelin, and water content within the neurological tissues [93]. Figure 2 compares the findings of between SWI, phase, and FLAIR imaging.

Furthermore, QSM can be used to evaluate deep grey matter (DGM), characterized by higher iron levels, which have been linked to increased susceptibility. This has been observed in several grey matter regions, including the caudate nucleus and globus pallidus, and could be related to increased neuronal damage [94]. Another study investigated the iron content of DGM structures over a two-year period found a significant decrease in iron content in the globus pallidus, caudate, thalamus, and putamen. The study also investigated the link between lower thalamic susceptibility and neurological disabilities, including physical abilities and cognitive tasks, and showed a significant association with increased hyperintensity T2 lesion volumes and decreased tissue volume in the entire brain, DGM, and the thalamic structure [95]. This could interpret the increased susceptibility as temporary changes associated with pathological changes during MS progression, such as brain atrophy or metabolic processes. This is a contradictory issue, as more longitudinal and multicenter imaging studies are required to better validate the results [96].

On the other hand, QSM has several limitations, which may pose technical challenges, such as post-processing QSM maps and removing background noise. Furthermore, clinical outcomes can be difficult to interpret. The calculated susceptibility values may differ depending on age, gender, or pathological changes occurring during the disease [96,97].

**Figure 3:**
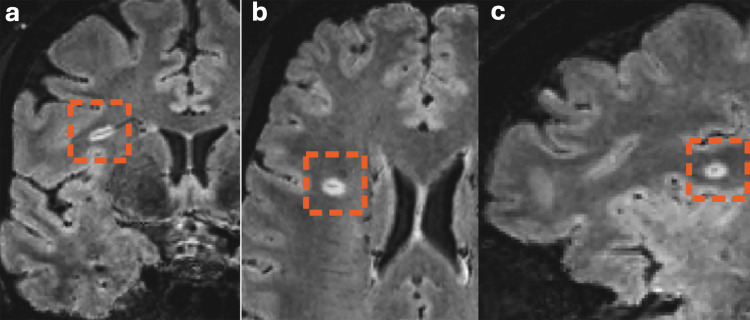
A zoomed-in (a) coronal, (b) axial, and (c) sagittal post-contrast T2 FLAIR* at 3T revealed CVS within the WM lesion indicated by the orange dotted rectangle. Reproduced from Rocca MA et al [3] under the terms of the Creative Commons Attribution License (CC BY 4.0).

Recent studies demonstrated the application of post-processing FLAIR* images, which result from the combination of T2 FLAIR and T2*-WIs. This has assisted in detecting more hyperintense MS lesions with hypointense CVSs [90,98]. Examples of central vein signs are illustrated in Figure 3. The diagnostic evaluation of the CVS is becoming more feasible with the development of automatic detection methods that are not significantly different from manual assessment [99]. In addition, the CVS boosts diagnostic accuracy once it is combined with other laboratory results, such as CSF oligoclonal bands (OCBs) [89].

Furthermore, the CVS was demonstrated in paediatric MS patients. In one study, the CVS was depicted with paramagnetic rim lesions (PRLs). In that study, 86% of the patients had at least three CVSs and more than one PRL within WM areas. This indicates the role of the CVS in both adult and paediatric MS populations [100].

The CVS is an important MS imaging biomarker; however, there are still limitations to its use. First, it is not exclusive to MS. Therefore, the assumption of other neurological findings cannot be excluded [101]. Second, there are requirements to establish protocols or rules to standardise detection methods. This is paramount for the acceptance of the CVS as a diagnostic imaging tool. Despite this, the 40% rule, which states that at least 40% of WM lesions with the CVS are probably MS lesions, produces high sensitivity and specificity, and has been applied successfully to MS paediatric patients [100].

Diffusion-Weighted Imaging (DWI)

Brownian motion can be described as the movement of water molecules within intra- and extracellular tissues, which could be restricted by cellular structure. Therefore, the water molecules have a defined direction of movement, which is known as anisotropic diffusion [102]. If the water molecules are randomly distributed and have no probable direction, this is defined as isotropic diffusion. To measure the diffusion of water molecules, diffusion gradients are incorporated into very fast MRI pulse sequences, such as echo planar imaging (EPI). This rapid sequence is paramount to measure the diffusion accurately and reduce the effect of other motion sources, such as breathing and other involuntary movement [103].

DWI is a recently developed routine in MRI protocols for imaging the brain. It can evaluate the brain at the microstructural level and has higher accuracy compared with other imaging sequences such as T2-WIs or FLAIR, with potential applications in evaluating stroke within minutes of onset [104]. In most clinical applications, diffusion gradients are applied in three orthogonal directions. This permits the construction of apparent diffusion coefficient (ADC) maps, which are diffusion-averaged to quantify the diffusion pattern. Areas where diffusion is restricted appear as bright signals on the diffusion-weighted (DW) images, in contrast to appearing hypointense on ADC maps, because ADC values are low. On the other hand, free-diffusing water presents as hypointense and hyperintense on DW images and ADC maps, respectively [103,105].

DWI with an EPI sequence is a rapid MRI technique; therefore, it is preferable during the assessment of MS lesions [106]. Both DW images and ADC are evaluated together for comparison and to gain a better understanding of lesion patterns. As DWI captures how water molecules move within tissues, the ADC map quantifies this movement, offering a numerical value that reflects tissue cellularity and integrity. Recent or acute MS lesions can be demonstrated as hyperintense signals on DW images and hypointense signals on the ADC map. This can be inferred as an imaging biomarker of the disruption of the blood-brain barrier [107]. Restricted diffusion can be observed with other pathological changes, such as during the early detection of stroke or brain abscess. Therefore, it is difficult to interpret restricted diffusionas an early MS change. While DWI cannot replace Gd-enhanced imaging agents, it might limit the use of contrast agents in MS imaging [108,109].

It is important to interpret ADC findings alongside other conventional MR images, either T2-WIs or post-contrast T1-WIs, as the ADC value will likely change based on the age of the acute MS lesions [110]. During the early stages of acute MS, there is a reduction in the ADC value, which correlates with a slight increase in T2-WIs and faint or zero enhancement on T1-WIs. Between 7 and 10 days, as the MS lesions become apparent, there is a high signal on T2-WIs and more enhancement on T1-WIs. ADC measurements then become pseudo-normal. As the acute MS lesions age, passing 4 weeks, there are marked increases in ADC values, which could be interpreted as vasogenic oedema [107].

In addition to ADC’s role in the assessment of acute MS lesions, it is promising in terms of monitoring MS treatment. It has been reported that the ADC values of acute and chronic MS lesions are significantly reduced compared with the pre-treatment ADC values. This suggests a role of ADC in tracking DMT [111]. In addition, an alteration in ADC values in normal-appearing white matter (NAWM) is a prognostic imaging biomarker. A 3-year follow-up study demonstrated the contribution of ADC to the loss of “no evidence of disease activity” (NEDA) status [112]. Despite the potential role of ADC as a biomarker, it is still under investigation due to the variability of ADC measurements across different MRI centres [113].

In most DWI setups in clinical applications, the diffusion gradients are applied in three orthogonal directions, which limits the accuracy in defining the diffusion direction and, consequently, how water molecules bounce. Diffusion tensor imaging (DTI) was introduced to overcome these challenges by performing DWI experiments in multiple directions [114,115]. At least six diffusion measurement directions are required to define the DTI matrix and, consequently, better track diffusing water molecules. DTI provides a variety of quantitative measurement maps that include axial diffusivity (AD), radial diffusivity (RA), mean diffusivity (MD), and fractional anisotropy (FA) [116]. AD quantifies how fast the water molecules are diffusing along the nerve fibres and evaluates the axonal integrity. RD is like AD but perpendicular to the neuronal axons and assesses the myelin integrity. In contrast, MD quantifies how the overall water molecules diffuse without directionality. FA is an index representing the amount of myelin content within the axons and ranges from 0 to 1. In the axonal area, where the myelin content is high, such as in the WM, the FA values approach 0.9 to 1. Therefore, if there is a reduction in the FA value, this could be interpreted as a demyelination of axons [103,117].

DTI is a unique imaging modality for evaluating WM structures because they are highly anisotropic, which means that they have a defined diffusion direction. In terms of MS diagnosis, DTI is a promising imaging tool for tracking all the microstructural changes in NAWM, a process that may not be feasible by other means [118]. DTI is very sensitive when evaluating this area and can identify abnormalities before MS lesions appear on a conventional MRI [119,120]. In addition, DTI can assist in the diagnostic workup because it is valuable for differentiating MS from other mimicking diseases; for example, MD values were reported to be significantly higher in MS compared with those in other lesions. This is valuable in determining a treatment strategy [121]. Acute MS lesions exhibited a decrease in AD and an increase in RD, that are related to acute axonal injury and myelin damage, respectively [111]. In chronic MS lesions, AD exhibited higher values and a decrease in RD values, which could be interpreted as increased extracellular space and greater axonal loss [122].

Quantitative DTI maps are very useful in terms of tracking disease progression after treatment is given either in NAWM or during the formation of new MS lesions [120,123]. A 4-year follow-up study demonstrated the role of DTI indices in predicting subtle changes in WM and how they are correlated with the development of neurological disabilities. It was reported that significant changes in the DTI quantitative maps of the corpus callosum occurred over the first year. However, these changes could not predict the development of disabilities over 4 years, and this study was limited to the corpus callosum and internal capsule [124]. FA quantitative maps provide indicators of the myelin levels in neuronal axons. FA values were low in MS black hole lesions, which indicating axonal damage in these MS lesions. In addition, similar changes in FA were demonstrated in NAWM. However, there was no significant difference between enhanced and hyperintense MS lesions [118]. A recent study reported an increase in AD and RD in acute MS lesions, which can be significant in predicting an evolving black hole [125].

Despite the role of DTI in evaluating subtle changes in NAWM, it has several limitations that require more attention, which could lead to the development of better imaging techniques. First, DTI cannot accurately resolve crossing and converging fibres or axons within a single voxel. Therefore, it may not be fully capable of evaluating the complexity of MS lesions [126]. Second, the reproducibility of DTI is low, and it requires more standardisation to provide accurate and reliable results. This has led to the development of advanced diffusion imaging techniques, such as diffusion kurtosis imaging, which provide better insight into the microstructural changes in the brain [127].

Future directions

Artificial intelligence (AI) plays an important role in current MRI research. This has been demonstrated by significant advances in AI technologies such as machine learning (ML) and deep learning (DL). This is reflected in MS MRI in terms of diagnosis, management, prognosis, and treatment planning strategies [128-131]. Manual lesion detection and segmentation is time-consuming and prone to error. Recent studies have shown the reliability of AI with automated assessment, such as detecting CLs at 3 tesla scanners and evaluating enhanced MS lesions [128,129]. Furthermore, this method was extended to assess advanced MRI biomarkers, such as the CVS and PRLs, which are frequently detected in the white matter [130-133]. Automatic assessment using AI technologies has improved the workflow in the MRI department by reducing workloads for reporting MS cases or making critical decisions, such as administering Gd contrast agents [134]. Another study used baseline MRI data to demonstrate the ability of ML with the XGBoost algorithm to predict how MS is progressing and its relationship with the EDSS [135]. Despite the promising applications of AI in evaluating MS lesions, there are some limitations that require further consideration, such as the need for large amounts of datasets, which are typically limited by ethical and policy concerns [136,137].

## Conclusions

There is no single imaging biomarker that can answer all critical questions relating to MS progression and the patient’s response to DMT. Radiology reports frequently include the lesion location, which is paramount for correlation with clinical symptoms or determining the efficiency of DMT. The detection and localization of enhanced and hyperintense MS lesions are indicators of disease activity and progression. Black hole MS lesions and brain atrophy are significant predictors of neurological disabilities in MS. With the development of MRI scanners, more cortical MS lesions can be detected, resulting in better MS diagnosis and prognosis. Advanced MRI biomarkers are promising but should still be combined with conventional biomarkers, which results in increased specificity and better interpretation of MS heterogeneity.
